# Where are all the egg genes?

**DOI:** 10.3389/fcell.2023.1107312

**Published:** 2023-02-03

**Authors:** Katherine A. Maniates, Andrew Singson

**Affiliations:** Waksman Institute of Microbiology, Rutgers University, Piscataway, NJ, United States

**Keywords:** egg, oocyte, fertilization, *C. elegans*, mouse, *zebrafish*, sperm

## Abstract

Complementary forward and reverse genetic approaches in several model systems have resulted in a recent burst of fertilization gene discovery. The number of genetically validated gamete surface molecules have more than doubled in the last few years. All the genetically validated sperm fertilization genes encode transmembrane or secreted molecules. Curiously, the discovery of genes that encode oocyte molecules have fallen behind that of sperm genes. This review discusses potential experimental biases and inherent biological reasons that could slow egg fertilization gene discovery. Finally, we shed light on current strategies to identify genes that may result in further identification of egg fertilization genes.

## 1 Introduction

### 1.1 The big questions and fertilization gene discovery

Understanding the genetic underpinnings of fertilization is essential for developing infertility treatments, contraceptive targets, understanding speciation, and mechanisms of cell-cell interactions (Schultz and Williams, 2002; Krauchunas et al., 2016; Bhakta et al., 2019; Findlay et al., 2019). In the last several years there has been a rapid increase in the discovery of genetically validated fertilization molecules in several key model systems (Deneke and Pauli, 2021; Mei and Singson, 2021). While fertilization is studied in many model systems, forward and reverse genetic approaches in worms, zebrafish, and mice have recently led the charge. Sterile mutants obtained through mutant screens or genetic knockouts are the modern gold standard to demonstrate the requirement of a gene to encode a factor in fertilization. The progress from genetic screens and knockouts has also established that there are fertilization genes that are deeply conserved from nematode worms to mammals (Mei and Singson, 2021). For example, there are several immunoglobulin superfamily molecules that were independently identified with roles in fertilization in several different species (Ellerman et al., 2003; Krauchunas et al., 2016; Binner et al., 2022). The DCSTAMP sperm molecules *spe-42* and *spe-49* (Kroft et al., 2005; Wilson et al., 2018) have essentially the same sperm sterile mutant phenotype to DCST1/2 mutants in flies, fish, and mice (Inoue et al., 2021; Mei and Singson, 2021; Noda et al., 2022). Progress in fertility gene discovery has also uncovered a high degree of molecular complexity required for sperm egg interactions. This was the inspiration for the concept of the fertilization synapse as the intellectual framework to understand how many newly discovered fertility gene products work in conjunction at the interface of sperm and egg plasma membranes (Krauchunas et al., 2016). Sperm and egg interactions have parallels with other cellular synapses (neural and immune) that include specialized cell structures, integrated with adhesion, secretion, and cell signaling. Understanding the fertilization synapse will require knowing what molecules interact either in cis with other molecules on the same gamete or in trans with molecules of the opposite gamete. The realization of the fertilization synapse also opens new questions. How are all the sperm and egg components assembled into complexes and at the right time and place? Genes are being discovered that may impact the processing and assembly of the fertilization synapse (Gleason et al., 2006; Contreras et al., 2022; Schreier et al., 2022). For example, Frey can regulate the assembly of Izumo1, a key protein involved in fertilization on the surface of the sperm (Contreras et al., 2022). It does this by helping to assemble Izumo into the correct protein complex. The road to the identification of genes involved in fertilization faces many challenges which researchers must overcome.

### 1.2 Difficulties in determining genes involved in fertilization

Unlike some other biological processes, fertilization comprises multiple cellular processes including gamete activation, recognition, binding, and fusion. These processes must be executed very precisely to combine one sperm and one egg. This requires that the space, time, and the concentration of proteins during fertilization be exact. The transient interactions and combination of proteins at the correct level can be difficult to recapitulate *in vitro*. Genes must be expressed specifically on the surface of the cell and often shift after fertilization. As there are multiple processes that require very specific interactions, it is of no surprise that the sperm and egg also require multiple protein interactions. Beyond the transient interactions, redundancy, pleiotropy, and maintaining sterile mutants have been roadblocks for gene discovery on both gametes.

### 1.3 Current fertilization molecules and open questions

**FIGURE 1:**
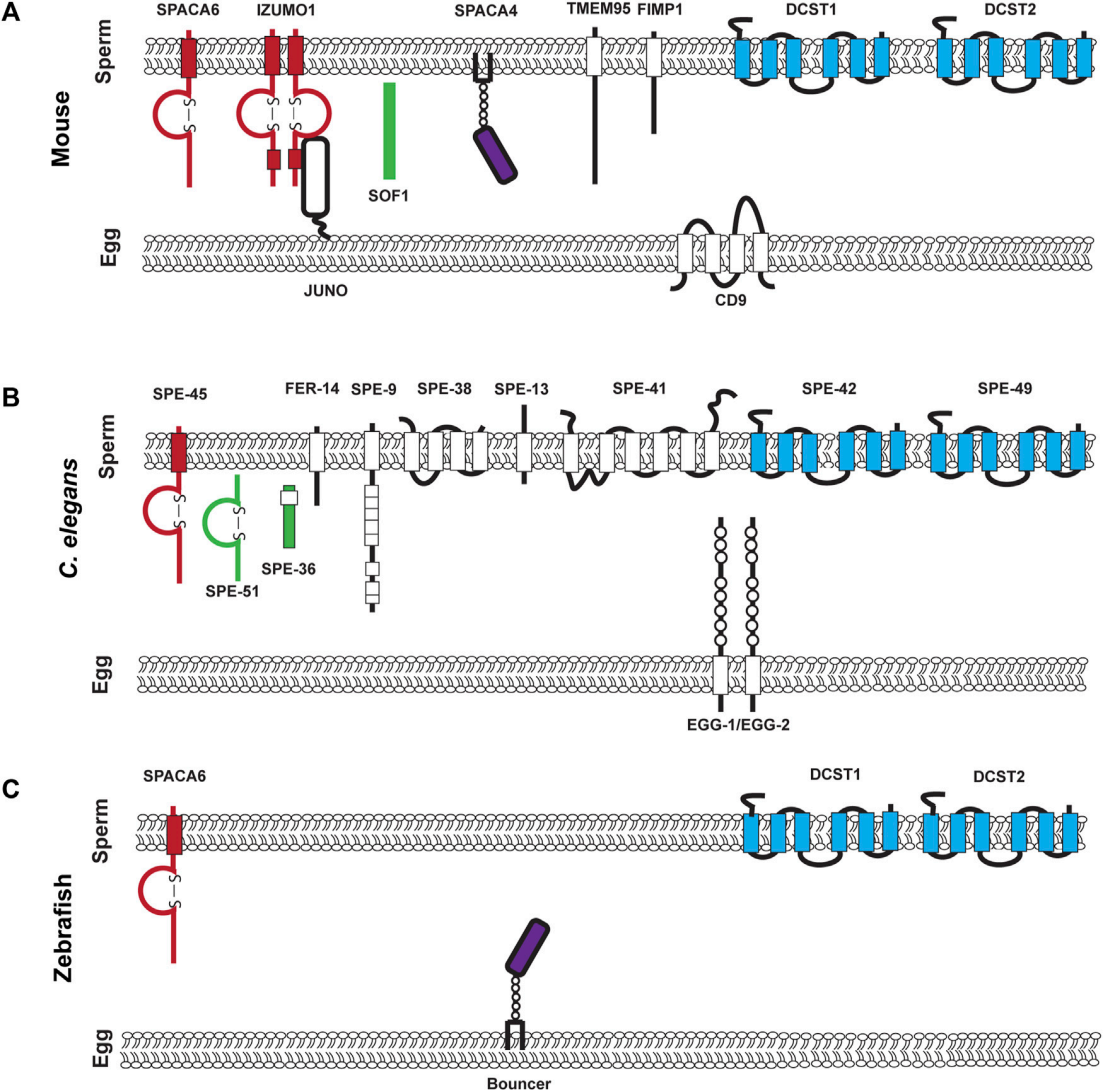
Known components of the mouse, *C. elegans*, and Zebrafish fertilization synapse. **(A)**. Mouse fertilization synapse, **(B)**. *C. elegans* fertilization synapse, and **(C)**. Zebrafish fertilization synapse. For **(A–C)** each of the molecules that are denoted have been experimentally validated though loss of function mutations. Proteins on the left of each synapse in red contain an immunoglobulin domain, proteins in green are secreted, proteins in purple are in the uPAR/Ly6 family and on the right in blue are conserved DCSTAMP proteins.

As of writing this review, going by the sterile mutant gold standard, there are ten mouse genes (Bianchi and Wright, 2020; Deneke and Pauli, 2021) and 12 *C. elegans* genes that encode transmembrane or secreted proteins that are required for fertilization and are components of the fertilization synapse (Mei and Singson, 2021) (Figure 1). For complete recent information about the fertilization proteins, please see recent reviews by Deneke and Pauli (2021); Mei and Singson (2021). Anyone would agree that when it comes to fertilization, it takes two to tango as sperm and egg are the ultimate in complementary cells. However, gazing at the molecules in the emerging fertilization synapse of worms, fish, and mouse shown in Figure 1 there is a striking asymmetry. Most currently known fertilization genes encode sperm factors. Ten of twelve genes in worms and seven of ten genes in mice are on the sperm surface (Figure 1). Here we review why we currently observe this gene discovery asymmetry between known sperm and egg genes. As of writing this review, only five egg surface molecules have been discovered: *egg-1/2* in *C.* elegans, Bouncer in Zebrafish, and Juno and CD9 in mouse (Naour et al., 2000; Kadandale et al., 2005; Bianchi et al., 2014; Herberg et al., 2018) (Table 1). These genes encode a multitude of different types of proteins. *Egg-1* and *egg-2* have LDL receptor repeats, Bouncer a Ly6/uPAR protein, Juno, is related to folate receptors, and CD9 is a tetraspanin (Naour et al., 2000; Kadandale et al., 2005; Bianchi et al., 2014; Herberg et al., 2018). However, these genes are not sufficient for all the different functions of fertilization. Therefore, the search for egg genes continues. There are other proteins such as Phospholipid C zeta which is a sperm specific soluble enzyme that can trigger oocyte activation (Yoon et al., 2008; Yoon et al., 2012; Hachem et al., 2017; Sanders et al., 2018). This review in particular focuses only on proteins that are expressed on the surface of the gamete. This asymmetry underscores the importance of studies in females. Female processes have been historically understudied. This has been a focus of the NIH since 2016 when they published their policy on sex as a biological variable (Arnegard et al., 2020). The gap in egg gene discovery is an important subject that we, as researchers, should examine and work to close. In this review, we discuss different biological mechanisms such as redundancy, pleiotropy, and how the evolution of sperm expressed genes have impacted the identification of fertilization genes in the oocyte. A likely source of lagging egg gene discovery is also experimental bias either in methods or the model organisms. Potential experimental biases could include maintenance and propagation of sterile mutants, screening strategies, and difficulty in identifying fertilization phenotypes. While many of these experimental biases are not an exclusive challenge to discovering egg genes, we think that these are important ideas to bring to the forefront of the research community. Finally, we conclude by discussing new experimental approaches and options that could address the question of where are all the egg genes?

**TABLE 1:** Egg surface fertilization molecules.

Gene	Species	Protein domains and features	References
*egg-1*	*C. elegans*	Transmembrane protein with LDL repeats	Kadandale et al. (2005)
*egg-2*	*C. elegans*	Transmembrane protein with LDL repeats	Kadandale et al. (2005)
Juno	Mouse	GPI anchored folate receptor	Bianchi et al. (2014)
CD9	Mouse	Tetraspanin	Naour et al. (2000), Bianchi et al. (2014)
CD81	Mouse	Tetraspanin	Tanigawa et al. (2008)
Bouncer	Zebrafish	GPI anchored, Ly6 Superfamily	Herberg et al. (2018)

## 2 Challenges in the identification of egg genes

### 2.1 Fertilization and redundant genes

Fertilization is an essential process and redundant genes can help protect against deleterious mutations. The same protection that redundancy provides may make it more difficult to identify egg genes with genetic approaches. For sexually reproducing organisms, loss of fertility is evolutionarily devastating, rendering animals unable to pass on their genes to future generations. One random mutation could render an animal sterile and subsequently bring their fitness level to zero. Therefore, like other genes that have essential functions, gene duplication and redundant copies can decrease the likelihood of extinction occurring over many generations. This has been observed across many different organisms in fertility and particularly in the previously identified egg genes. In *C. elegans, egg-1* and *egg-2* function redundantly with one another. The loss of *egg-1* which encodes an LDL receptor repeat-containing protein that functions redundantly with *egg-2* another LDL receptor. These genes encode a protein with 67% similarity (Kadandale et al., 2005). The initial observation by RNAi of *egg-1* also knocks down *egg-2* generating a sterile phenotype, however genetic loss of only *egg-1* or *egg-2* decreases fertility but does not cause sterility.

On the oocyte surface, one abundant group of proteins that were at the forefront of fertilization research for many years were integrins on the egg surface. Interest in this group of proteins arose due to a disintegrin domain in ADAM2/Fertilin that was found on the sperm surface (Blobel et al., 1992). Investigation of this class of integrin proteins which were expressed in eggs revealed 24 different integrin combinations. The complexity made this a difficult area to study (Evans, 2002). Individual knockouts of integrins generated modest decreases in fertility and multiple knockouts generated other fertility defects such as embryonic lethality making this challenging to elucidate their exact role during fertilization (Miller et al., 2000; Hynes, 2002; He et al., 2003; Vjugina and Evans, 2008). While redundancy is not the singular reason that it has been difficult to identify egg genes, we hypothesize that it is one of the most prevalent reasons.

On the sperm side of the equation, there have also been genes that act redundantly with one another. One example is the CRISP (Cysteine-RIch Secretory Protein) gene family which regulates calcium channels in fertilization. In mouse, there are four CRISP family members, knockout of one member of the family reduces fertility but does not completely abolish it, however knockout of three or four of the family members completely disrupts male fertility demonstrating their redundant or compensatory functions (Ros et al., 2008; Curci et al., 2020). Interestingly in humans there are three CRISP genes which are all located on the same chromosome, therefore it would take a large deletion or rare collection of point mutations in all three genes to remove all function rendering them infertile (Gibbs et al., 2008; Curci et al., 2020). Redundant or compensatory genes may be a big challenge in our field in discovering egg genes as well as additional sperm genes. With the rise of CRISPR/Cas9 gene editing, it may be valuable to look for homologues and potential gene families and then knockout multiple genes in these family groups to examine for fertility phenotypes.

### 2.2 Less gene expression and pleiotropy

Oocytes enter a period of transcriptional quiescence usually during which fertilization, egg activation, and early embryonic development must occur (Kim and You, 2022). This contrasts with sperm cells which have a short period of quiescence, however their function largely ends after fertilization. The oocyte must provide all the proteins necessary for these very diverse cellular processes. One way that this could be accomplished is through pleiotropy, where one gene regulates multiple functions. As the first genomes were sequenced, researchers were surprised that there are far fewer genes than previously predicted, pleiotropy is one explanation for this phenomenon (Lander et al., 2001).

A related reason that it might be difficult to identify egg genes is that there may be tissue or stage specific isoforms of genes. This is where one isoform of a gene functions in development and another during adulthood, or one isoform in a somatic cell and other functions in the germline. For example, Juno in mouse is widely expressed in tissues other than just oocytes including the thymus, spleen, and lung however loss of Juno in these tissues does not appear to solicit additional loss of function phenotypes (Spiegelstein et al., 2000; Bianchi et al., 2014). If multiple phenotypes are exhibited this can complicate our ability to interpret gene functions. For example, in *C. elegans, nhr-23* is expressed during the process of molting which takes place during larval development and precedes much of germ cell differentiation. Later in development *nhr-23* also functions as a transcription factor during spermatogenesis (Ragle et al., 2020). The earlier molting defects exhibited by *nhr-23* had been established for many years prior to its function during spermatogenesis. The function during spermatogenesis was not as evident as animals arrested prior to adulthood (Kostrouchova et al., 1998; Kostrouchova et al., 2001). Finally, as mentioned before in Section 2.1, loss of integrins on the egg surface identified pleiotropic functions in oogenesis and embryonic development. Mutant phenotypes that are exhibited in other tissues or earlier in development can make the isolation of specific genes involved in fertilization more difficult and these pleiotropic defects may obscure specific genes roles in fertilization. The limited number of genes that are expressed in the oocyte make this a compelling reason why egg expressed gene roles have been difficult to identify.

### 2.3 Differential cell expression, opposing sex specific expression patterns, and homotypic interactions can make elucidating gene function difficult

Another reason that identifying egg genes could be difficult is that different proteins or protein domains maybe used in various species. Further it may be where two identical structural elements of individual monomers are interchanged to stabilize protein complexes (Bennett et al., 1994). The puzzle of molecular swapping between two gametes underscores the importance of studying fertilization in different organisms to understand the mechanism of cell-cell interactions between sperm and oocytes.

For example, Bouncer, a Ly6/uPAR protein in Zebrafish is essential for sperm-egg interactions which facilitate sperm binding to the oolemma when expressed on the egg surface (Herberg et al., 2018). SPACA4, the mammalian homologue of Bouncer is actually expressed on the sperm surface in mice and functions to penetrate the Zona Pellucida (ZP) for binding to the egg surface in mouse (Fujihara et al., 2021). At this point, the mechanism underlying opposing sex specific expression patterns for Bouncer and SPACA4 is not clear. Hypotheses surrounding this include it being due to different environments for fertilization as Zebrafish have a micropyle and therefore the sperm do not need to pass through the egg surface whereas mammals need penetration of the ZP and the acrosome reaction to occur. Another hypothesis is that this is a sperm specific loss of function that was retained in the egg during evolution.

In the same vein, homotypic interactions where either two proteins or protein domains interact with one on each of the opposing sperm and egg cells acting to signal for binding or fusion to occur. No homotypic interactions have been observed in fertilization thus far. However, this has been observed in cadherin signaling for polarity in *Drosophila* (Chen et al., 2008). Polarity is also required for fertilization and establishing egg activation. Another example related to cell-cell fusion is EFF-1 in *C. elegans* which mediates cell-cell fusion in the Soma (Segev et al., 2018). The EFF-1 interactions are structurally homologous to HAP2/GCS1 in flowering plants which is a sperm-egg fusogen in plants (Brukman et al., 2022). Homotypic interactions and sex specific expression patterns can make it difficult for identification of egg molecules as homotypic mutant animals would be sterile in both sexes and be very difficult to generate and maintain.

### 2.4 *De novo* gene formation in the testis and the co-evolution of fertilization receptors create complex genetic dynamics

Gene duplication and deletion events often drive evolution and establish new biological functions and phenotypes. Interestingly, it has been shown that *de novo* gene synthesis occurs much more often in the male lineage particularly through the testes (Kondo et al., 2017). This may also be referred to as the out of the testis hypothesis. It can be hypothesized that the abundant number of new genes in the male lineage may provide opportunities for these newly synthesized genes to be adopted into fertilization synapse complex working to stabilize this process and further define speciation. The male germline has several attributes that can facilitate new gene synthesis. These include histone modifications, demethylation of CpG islands, and increased expression of transcription associated proteins, as well as increased selective pressure from sperm competition (Kleene, 2001; Haerty et al., 2007; Kondo et al., 2017; Nyberg and Carthew, 2017). In contrast to gene synthesis, in gene loss, genes which no longer have biological functions can occur. Gene loss is less likely to occur in oocytes and primarily impacts multi-copy gene families (Assis, 2019). These results may demonstrate that while over time more sperm surface genes may be involved in fertilization, the genes on the oocyte side of the equation are less likely to change and may have strong levels of conservation and be involved in multiple processes such as both oogenesis and fertilization. It has also been hypothesized from observations in marine invertebrates that there is a co-evolution “arms race” of the male and female fertilization proteins in different species which may drive species specificity and could make identification of conserved fertilization molecules more difficult to identify (Wilburn and Swanson, 2016). The long-term evolutionary consequences of these changes in genes can both drive speciation and impact the genes that are involved in fertilization. These, however, are unlikely to be phenotypes that can be picked up in genetic screens but would require analysis of different species and genomes.

### 2.5 Sterile animals require active maintenance in the lab

As anyone who works in fertilization and gamete activation knows, maintaining mutant animals that are sterile or have lethal mutations requires active thought and coordination by researchers for each generation. To overcome these difficulties, there are a few very clever ways to maintain sterile animals for experimental analysis. One example is the identification conditional mutations. These conditional mutants can be temperature sensitive (ts) mutations that can be more easily screened for in microbes, worms, and flies. These ts animals are sterile when cultured at high temperatures but fertile when cultured at lower temperatures (also see Section 2.7 below) (Suzuki and Procunier, 1969; O’Connell et al., 1998; Mei and Singson, 2021). A second approach is through inducible systems such as Cre-Lox, GAL4/UAS, and Auxin Inducible Degrons which use either site specific recombinases, DNA binding activation sites, or chemical induction to induce sterile phenotypes (Brand and Perrimon, 1993; Zhang et al., 2015; Kim et al., 2018). Finally, we can use balancer chromosomes which are rearranged chromosomes often with morphological and fluorescent tags which can be used to maintain recessive lethal or sterile mutations as heterozygotes without recombination (Dejima et al., 2018; Miller et al., 2019). Unfortunately, all of these techniques have their limitations. Temperature sensitive mutations rely on protein misfolding at differential temperatures and there are relatively few model organisms where this is a practical approach (body temperature conforms to ambient temperature). Furthermore, not all genes are able to be mutated to become temperature sensitive, this favors proteins with hydrophobic residues and higher free energy levels (Varadarajan et al., 1996). Inducible systems require knowledge of the specific gene and favor reverse genetic approaches which can bias gene identification. Some balancer chromosomes rely on active maintenance of mutant animals as heterozygotes. Finally, collecting enough mutant animals for analysis may require specific breeding schemes as in the case of mice and the amount of space and resources required can limit the amount of exploration that can occur. For egg surface genes, extra care must be taken in order to maintain a set of fertile-heterozygotes in order to generate mutants for the next generation. This sibling selection may require constant costly and laborious molecular genotyping.

### 2.6 Gametogenesis and fertilization are often inherently temperature sensitive, egg genes might be more specifically unable to become temperature sensitive

One way that researchers working on worms, flies, and fish have managed to keep sterile animal lines going is through temperature sensitive mutations as described in Section 2.5 (Mei and Singson, 2021). While this has been an extremely useful tool, not all proteins can be mutated to become temperature sensitive. In fact, spermatogenesis and sperm activation are inherently more temperature sensitive than egg processes (Nakamura et al., 1987; Kurhanewicz et al., 2020; Hirano et al., 2022). The ability of oocytes to buffer temperature may make it more difficult to identify mutations but may also reveal information about the biology of oocytes. In many male-female organisms, oocytes are available in a limited number, therefore protecting the viability of oocytes becomes essential for an animal’s reproductive success, consequently our techniques of temperature stress may not be as efficient for egg specific genes.

### 2.7 Egg fertilization genes may be mischaracterized as embryonic lethal and meiosis mutants

Despite the difficulties in maintaining sterile animals, the field still engages in both forward and reverse genetic screens identify additional genes involved in fertilization. This has often required creative techniques such as temperature sensitive mutants, sibling selection, balancing all mutants prior to analysis, and using CRISPR/Cas9 to knock out all candidate genes to capture sterile alleles for future analysis (Mei and Singson, 2021). After mutagenesis one limitation is that it can be difficult to differentiate between genes that are embryonic lethal or have meiosis defects from fertilization specific genes at a high level since they often display similar terminal phenotypes. This can lead to a bottleneck in assessing mutations in a screen. Furthermore, on the flipside, when researchers are conducting screens for other phenotypes, they may encounter sterile mutants but not have the experimental tools to keep these mutations from getting dropped out of the population. It is possible that the egg surface genes that we are interested in may have been discarded in screens looking for embryonic lethal or meiosis genes.

### 2.8 Laboratory environments may not mimic environmental conditions that the animal might experience in the wild

Labs are often sterile environments that researchers work hard to keep free from contamination. However, this does not mimic the natural environment that our experimental organisms experience in the wild. Animals in laboratory environments do not face competition for food, contamination by parasites and bacteria, the temperature is stable, and light-dark cycles are controlled. These environmental stresses can often decrease fertility in the wild through changes in immune response, diet, and seasonality (Amaral et al., 2014). Therefore, in laboratory environments, we may be unable to recapitulate the environmental stressors that may impact fertility by modulating gene responses.

### 2.9 Egg fertility proteins may be sensitive to one mutant copy and quickly become sterile

In contrast to the idea that we’re unable to capture egg genes due to redundancy where there is no observable phenotype because there’s another copy of the gene which is compensating for the loss of one copy is a dominant sterile mutation. In a dominant sterile mutation, the loss of one copy of a gene through mutation renders an animal sterile or lethal (Erdélyi and Szabad, 1989). Capturing dominant sterile mutations may occur in genetic screens but lead to a dead end. It is incredibly difficult to keep dominant sterile strains alive. Maintaining a dominant sterile mutation requires the gene to have sex restricted expression where one sex is not sterile. For example, a dominant negative sperm mutation would not impact females, therefore the mutation could be carefully maintained in the maternal lineage and then crossed to a wild-type male and analyze their male progeny which would have one copy of the gene. One example of this is *wee-1.3* in *C. elegans*. The *wee-1.3* gene encodes a kinase that functions during spermatogenesis, a dominant mutation in this gene renders an animal infertile (Lamitina and L’Hernault, 2002). The hermaphrodite-male androdiecious reproductive mode in *C. elegans* is the only way that these mutant animals were able to be maintain. These limitations therefore preclude anything, that is, homotypic and expressed in both gametes. If prepared, it is possible to generate conditional mutants, however this excludes forward genetic screens and simple knockouts for analyses.

### 2.10 Small proteins are less likely to be mutated in genetic screens and less likely to be identified with biochemical methods

In fertilization small genes (less than 200 amino acids) have been shown to have important roles for fertilization. In Zebrafish, Bouncer, the Ly6/uPAR protein on the egg surface is 80 amino acids (Herberg et al., 2018). On the sperm side of the equation, *spe-13* in *C. elegans* is 130 amino acids (Singson Lab unpublished observation) and *fimp-1* in mouse with 111 amino acids are also small proteins involved in fertilization (Fujihara et al., 2020). Despite their important roles, small proteins are more likely to be missed in both genetic screens as well as mass spectrometry or ribosomal profiling (Jorgensen and Mango, 2002; Harney et al., 2019). In genetic screens the smaller number of nucleotides in each gene decreases the probability that individual base pairs will be mutated in the right region for sterility to occur. For mass spectrometry and ribosome profiling, this is also an issue as RNA based methods are more likely occurring with amplified RNA which can filter out smaller RNAs, and small transcripts may be beyond the sensitivity of the equipment (Ahrens et al., 2021). The emergence of the importance of small proteins may be a good avenue for future analysis.

### 2.11 Mutations in egg fertilization genes may generate extremely subfertile animals but not be labeled fertilization defects

The question of what constitutes a sterile phenotype may also mire the picture of what genes are involved with fertilization on the egg’s surface. Infertility is clinically defined as failure to conceive after 12 months of unprotected intercourse (Mélodie and Christine, 2018). This also discounts couples that may be able to have one child but then experience secondary infertility. A significant drop in the number of progeny that an animal produces due to a genetic mutation while not completely sterilizing an animal could make it difficult to determine if involvement is technically in fertilization. There is some debate in what should be considered subfertile or sterile, particularly in animals with larger broods and faster rates of ovulation. For example, mice ovulate at 8 times the rate of humans with more than one egg at a time, a decrease in litter size here could look like clinical infertility in humans (Vjugina and Evans, 2008). Similarly in *C. elegans* which have a brood size of ∼300 progeny in their reproductive lifetime, a decrease to two progeny in their lifetime would be very likely to be considered clinically infertile if modeled in humans. Thus, we encourage careful analysis of animals with fertility defects and a careful analysis of the phenotypes in determining their role at the molecular level.

### 2.12 Previous biochemical analysis has often been unable to be genetically validated

Previous biochemical analysis primarily in sea urchin and abalone identified a multitude of proteins such as Bindin, speract and resact, VERL and lysin through analysis of cell extracts using monoclonal antibodies to inhibit fertilization (Vacquier and Moy, 1977; Hansbrough and Garbers, 1981). These approaches were valiant and groundbreaking to our understanding of protein candidates for fertilization. However, when genetic knockouts were examined for many of these genes, the animals were still fertile (Deneke and Pauli, 2021). Monoclonal antibodies to gamete surface antigens were helpful in identifying and validating IZUMO (Okabe et al., 1988). CD81 was also identified through monoclonal antibodies but is not essential but still can contribute to fertilization (Takahashi et al., 2001; Rubinstein et al., 2006). As we continue our search for all the genes that are involved in fertilization these contributions help us to understand the redundancy of the process and the contributing factors.

### 2.13 Historical narratives surrounding the roles of females may impact current egg gene discovery

Both historically and currently, females are understudied (Ah-King et al., 2014). Despite awareness and advocacy of this issue from individual researchers to as well as organizations such as the NIH, this gap has persisted (Arnegard et al., 2020). Commonly described reasons for why females continue to be understudied include that there is a stronger interest in male processes of reproduction, and that eggs are presumed to be less complex and passive receivers of sperm whose biology has already been fully characterized (Méndez and Córdoba-Aguilar, 2004). These reasons are not evidence based and fail to take into account recent gene discovery in fertilization, egg activation, and blocks to polyspermy which still have largely unknown mechanisms and complex cell signaling and organization. Perpetuating these ideas minimizes the role of females and can be potentially harmful in the treatment of female reproductive health.

## 3 Addressing the sperm and egg gene discovery question

To address the difficulty in identifying egg genes (and in fact sperm genes), there are several approaches that can be and are currently being taken (Table 2). For example, in *C. elegans* we are employing an approach which will involve doing random mutagenesis and then using balancer chromosomes to generate stable balanced lines of mutants, in contrast to previous screens which have identified temperature sensitive mutants that are typically not genetic nulls. This approach in invertebrate systems can allow for higher throughput of mutations to be screened as well as help differentiate embryonic lethal and meiosis mutants. In parallel with characterization of genetic mutants, Whole Genome Sequencing (WGS) can be employed to identify the causative mutation. Another approach being undertaken is to use CRISPR-Cas9 to generate knockouts of all testes expressed genes as is being undertaken by several labs to ascertain which knockouts impact fertilization (Miyata et al., 2016; Abbasi et al., 2020). A similar approach could be taken to knock out all oocyte expressed genes. This is however an expensive approach and can miss redundant or pleiotropic genes. Redundancy and pleiotropy remain problems which are difficult to address. A bioinformatic approach identifying potential gene pairs could aid in prioritizing generating double mutants could partially address this issue. Finally, for pleiotropic genes, conditional systems such as those mentioned in Section 2.6 can be employed. One caveat to this approach is that it is limited in the number of candidates that this would be feasible for as these can be labor intensive strains to build. Complementary to these approaches, utilizing biochemical approaches such as immunoprecipitation and proximity labeling will help us to better understand other proteins that are involved in fertilization. Taking advantage of biochemical approaches now supported by complementary genetic approaches will bring all available tools to bear on our understanding of how nature does conception.

**TABLE 2:** Solutions to identifying egg genes.

Unbiased forward genetic screens utilizing genetic tools such as balancer chromosomes to identify non-conditional mutants
Systematic knockout of all oocyte expressed genes and gene pairs using CRISPR/Cas9
Immunoprecipitation and proximity labeling to identify candidates followed by genetic validation

## 4 Conclusion

Is this question of where are all the egg genes an experimental problem or a true biological asymmetry? We imagine that it might be a combination of both possibilities. Will this asymmetry of sperm and egg molecules also be seen in other species such as fish, frogs, and humans? We have great faith in the determination and productivity of the reproductive biology community to continue with its rapid and transforming fertility gene discovery. Exciting new experimental approaches to aid in egg gene discovery are rapidly coming into play. The future discoveries of egg genes will greatly enhance our understanding of fertilization to aid in the development of infertility treatments and novel contraceptive techniques as well as provide further understanding of how two morphologically distinct cells interact with each other.
